# Identification of strengths and weaknesses of the healthcare system for persons living with rare diseases in Catalonia (Spain), and recommendations to improve its comprehensive attention: the “acERca las enfermedades raras” project

**DOI:** 10.1186/s13023-024-03518-x

**Published:** 2025-01-29

**Authors:** José Hernández-Rodríguez, Fernando Martínez-Valle, Xènia Acebes, Carmen Alerany, Jordi Antón, Gonzalo Calvo, Marian Corral, Jordi Cruz, M. Antònia Mangues-Bafalluy, José Mateo, Josefa Rivera, Albert Salazar, Roser Francisco, Cristina Mallol, Rita Reig-Viader, Ariadna Tigri-Santiña, Assumpta Ricart, Francesc Palau

**Affiliations:** 1 Clínic Program for Rare Diseases, Department of Autoimmune Diseases, Hospital Clínic de Barcelona, Institut d’Investigacions Biomèdiques August Pi i Sunyer (IDIBAPS), University of Barcelona, Barcelona, Spain; 2 Division of Systemic Autoimmune Diseases, Internal Medicine Department, Hospital Universitari Vall d’Hebron, Barcelona, Spain; 3 HealthCare Management Department, Hospital de la Santa Creu i Sant Pau, Barcelona, Spain; 4 Pharmacy Department, Hospital Universitari Vall d’Hebron, Barcelona, Spain; 5 Pediatric Rheumatology Department, Hospital Sant Joan de Déu, Department of Surgery, Medical-Surgical Specialties and Pediatrics, University of Barcelona, Institut de Recerca Sant Joan de Déu, Barcelona, Spain; 6 Department of Clinical Pharmacology, Area del Medicament, Hospital Clinic de Barcelona, Institut d’Investigacions Biomèdiques August Pi i Sunyer (IDIBAPS), Barcelona, Spain; 7 Asociación Española de Laboratorios de Medicamentos Huérfanos y Ultrahuérfanos (AELMHU), Barcelona, Spain; 8 FEDER (Federación Española de Enfermedades Raras) Foundation, Madrid, Spain; 9 MPS-Lisosomales Association, Barcelona, Spain; 10 Pharmacy Department, Hospital de la Santa Creu i Sant Pau, Barcelona, Spain; 11 Thrombosis and Hemostasis Unit, Department of Hematology, Hospital de la Santa Creu i Sant Pau, Barcelona, Spain; 12 Rare Diseases Unit, Parc Taulí University Hospital, Sabadell, Barcelona, Spain; 13 Health Services Research Group, General Management, Hospital Universitari Vall d’Hebron, Barcelona, Spain; 14 Rare Diseases Program, Catalan Health Service and Ministry of Health, Generalitat de Catalunya, Barcelona, Spain; 15 Integrated Health Processes Department, Healthcare Area, Catalan Health Service, Barcelona, Spain; 16 Laboratory of Neurogenetics and Molecular Medicine, Center for Genomic Sciences in Medicine, Institut de Recerca Sant Joan de Déu, Únicas SJD Center, Hospital Sant Joan de Déu, Barcelona, Spain; 17 Centro de Investigación Biomédica en Red de Enfermedades Raras (CIBERER), ISCIII, Madrid, Spain; 18 Division of Pediatrics, University of Barcelona, Barcelona, Spain

**Keywords:** Rare diseases, Person living with a rare disease, Comprehensive care, Patient journey, Healthcare system, Focus group

## Abstract

**Background:**

Rare diseases (RDs) are a heterogeneous group of complex and low-prevalence conditions in which the time to establish a definitive diagnosis is often too long. In addition, for most RDs, few to no treatments are available and it is often difficult to find a specialized care team.

**Objectives:**

The project “acERca las enfermedades raras” (in English: “bringing RDs closer”) is an initiative primary designed to generate a consensus by a multidisciplinary group of experts to detect the strengths and weaknesses in the public healthcare system concerning the comprehensive care of persons living with a RD (PLWRD) in the region of Catalonia, Spain, where a Network of Clinical Expert Units (Xarxa d’Unitats de Expertesa Clínica or XUEC) was created and is being implemented since 2015. The additional primary aim was to propose recommendations to solve or improve the limitations found.

**Methods:**

A task force of 13 participants with multidisciplinary expertise on RDs completed a questionnaire and participated in two focus groups. A document was drafted with an item series of strengths and weaknesses of the healthcare system regarding the care of PLWRD, and a set of proposals or recommendations to overcome the problems identified.

**Results:**

The Catalan Government healthcare model of XUECs for the comprehensive care for RDs is currently valid and adapted to the needs of PLWRD and their families since its strategic optimal and operational framework, and it is aligned with the European Reference Networks (ERNs) thematic areas. The problems found in the current healthcare model were grouped into ten main areas: (1) the healthcare model for RDs; (2) coordination with primary healthcare providers and other tertiary and secondary hospitals; (3) access to and coordination with non-medical services; (4) the role of case manager in the XUEC; (5) genetic diagnosis; (6) undiagnosed patients; (7) treatments; (8) referring process, continuous follow-up, and transition from pediatric to adult centers; (9) research and education for professionals; and (10) associations of PLWRD and their families (patients’ advocacy). The need for more resources was currently detected as the common factor for most of them. Ten key recommendations to improve the healthcare system regarding RDs were postulated.

**Conclusions:**

Catalonia has established a unique healthcare model for RDs in Spain, with clear strengths and advantages. However, after analyzing them, the experts suggested that new governmental political and administrative decisions are needed to ensure the efficient implementation of a healthcare plan for PLWRD in Catalonia, which could be applied to other regions and nations worldwide.

## Background

The term “rare disease” (RD) was first used in the 1980s in the United States in the field of hereditary metabolic diseases [1]. This term is closely related to the term “orphan drugs” and both were developed simultaneously to address the challenges of diseases with very low prevalence [1, 2]. Over 7000 RDs have been identified, of which 65% are severe and disabling and not all patients have some diagnostic technology or treatment available [3]. Roughly, 43% of individuals affected by these diseases lack access to any kind of therapies or receive inadequate treatment [4]. In the European Union (EU), RDs refer to those that affect fewer than 5 individuals in 10,000 inhabitants [5]. In Spain, it is estimated that RDs impact between 6 and 8% of the population at any point of their lives, affecting approximately 3 million people in the entire country [6]. In Catalonia, an autonomous region in Northeastern Spain with a population of 8 million, the estimated prevalence of rare diseases ranges from 300,000 to 400,000 patients [7].

RDs are characterized by a wide range of clinical manifestations that vary from disease to disease and from patient to patient. Due to the low prevalence of each disease, medical expertise, knowledge, care offerings, and research are all limited [8]. However, the real impact of RDs extends beyond their clinical care, since patients often have additional social, educational, occupational, and family needs. These concomitant factors transform RDs into not only medical problems, but also significant issues with social and economic implications [4, 9].

Persons living with a RD (PLWRD) usually face unique challenges, such as long-lasting diagnosis times or physicians and healthcare providers (HCPs) with limited experience in identifying, diagnosing or managing RDs. Evidence worldwide supports these facts, as the process of diagnosis of a RD is lengthy and many patients usually have to endure interactions with multiple specialists and HCPs to be accurately diagnosed during what is called patient’s journey or patient’s odyssey [10]. In fact, it is estimated that a PLWRD in Europe spends between 5 to 30 years to obtain a diagnosis, a quarter of patients need to travel to a different region to be diagnosed, and more than 40% of them are misdiagnosed multiple times [10]. Experts also estimate that, in more than 30% of cases, the molecular origin has not been identified after following the established diagnostic protocols [11]. Once the diagnosis is made, the next hurdle experienced by patients is finding an expert physician or team to perform an adequate follow-up of the disease, as well as finding information about the disease, including its clinical course, disease complications, and appropriate therapies and centers with ongoing clinical trials, because they usually do not exist or are not available in their region [10]. In many cases, such information is hardly available, as not much research is usually performed on RDs [12]. In this sense, the Orphanet nomenclature and codification of RDs, which is fundamental for improving the visibility of RDs in healthcare, data processing and research, has positively evolved during last years, and even a new code for undiagnosed diseases (ORPHA: 616874, for “rare disorder without a determined diagnosis after full investigation”) has been recently generated [13].

Treatment options for PLWRD (whenever they have) tend to be orphan drugs, which are defined as new, innovative, and usually biotechnological agents for patients who have no other therapeutic alternatives [14]. Among the 199 orphan drugs with trade names in the European Medicines Agency (EMA) by December 2023, 147 have been approved for marketing in the European Union. Although 123 orphan drugs had a National Code in Spain, only 78 of them were being funded, with a mean governmental resolution time of the funding decision of 23 months [3]. In other countries, there are options that facilitate early access, even to patients, to innovation in areas where there is a clear unmet therapeutic need [15].

Organizations or associations of PLWRD have proved to make meaningful contributions to RDs healthcare such as advocating for new (orphan) drugs approval and reimbursement processes, providing psychological support to patients and families, or by giving them advice on the risks and benefits of certain therapies, among others [16]. Patient associations or advocacy groups should be part of decision-making forums as a way of incorporating patient experiences and outcomes into drug evaluation and health outcomes measurement. EURORDIS, the alliance of PLWRD advocacy groups, represents more than 1,000 patient associations in 74 countries [17] in European healthcare and pharmaceutical policy forums. At national and regional level, PLWRD are also increasingly involved in decision-making, which contributes to build a truly patient-centered system [17]. The Spanish Federation of RDs (Federación Española de Enfermedades Raras or FEDER) and the Catalan Federation of RDs (Federació Catalana de Malalties Minoritàries or FECAMM) are two federations of patient associations playing an active role in the Spanish and Catalan governments’ actions, respectively.

Within the European Union (EU), several actions have been promoted, aimed at improving the quality of healthcare, acting either at the regional, member state or European level. The Ministry of Health of the Generalitat de Catalunya (the Government of Catalonia) is in charge of matters of public health, including the control and coordination of the healthcare centers and legislation on pharmaceutical products. The Catalan Health Service or CatSalut (Servei Català de Salut,) is a public institution attached to the Ministry of Health, integrated into the Spanish National Health System (NHS). In 2009, the CatSalut created the Advisory Commission on RDs (Comissió Assessora de Malalties Minoritàries or CAMM) to develop an integral care model for RDs and its implementation in the region. In 2010, a new model for RDs care was defined to respond to the health, social and educational needs, and to improve diagnostic, continuous healthcare and therapeutic aspects of PLWRD [7]. The Catalan model mirrors the European Reference Networks (ERNs) model, and is based on the creation in 2015 of the Networks of Clinical Expert Units (Xarxes d’Unitats d'Expertesa Clínica or XUECs), in which all clinical specialized units have demonstrated experience and knowledge on the diagnosis and treatment of different groups of RDs [18]. All the accredited and designated units have to fulfill the quality criteria previously established by the CatSalut through supervised working groups of experts in every group of RDs. All the designated units belonging to a XUEC must share and promote clinical and scientific experience and knowledge about care of the specific groups of RDs, and also to work in a coordinated manner and with common standardized clinical guidelines or protocols. Additionally, they have to collaborate with territorial care resources including primary and community care providers, mental health services, intermediate care, among other complementary departments [7].

In Spain, the NHS created in 2006 the Reference Centers, Services and Units (in Spanish: Centros, Servicios y Unidades de Referencia or CSUR) in order to identify national reference units in tertiary hospitals specialized in complex and rare diseases, but also in the performance of specific medical or surgical procedures [19, 20]. The ERNs were created as virtual networks in 2017 of HCPs from the EU Member States and countries of the European Economic Area to provide effective, efficient, and high-quality care to complex or low-prevalence diseases requiring a concentration of resources or expertise by achieving cooperation between countries [21–24].

The project “acERca las enfermedades raras” (in English: “bringing RD closer”) is an initiative that aimed to offer solutions that improve the quality of life of PLWRD by a task force of experts. The main objective of this project was to detect the strengths and weaknesses of the Catalan Health Service regarding RDs and to propose subsequent recommendations to address the problems identified in the comprehensive care of PLWRD in Catalonia.

## Methods

### Context and characteristics of the task force

The task force consisted of a panel of 13 experts on different RDs fields aiming to identify the strengths and weaknesses of the healthcare system in Catalonia regarding RDs by completing a questionnaire and participating in two focus groups.

Ten out of the 13 participants were multidisciplinary specialized physicians (in clinical genetics, internal medicine, pediatrics, hematology, pharmacy, and clinical pharmacology) from five reference university hospitals in Catalonia as HCPs attending PLWRD. The three remaining members included a hospital manager and two representatives from the association of biotech/pharmaceutical companies and from FEDER patients’ alliance, respectively. Several experts were also involved in the coordination of Catalan (XUEC), Spanish (CSUR) and European (ERN) networks, or the Spanish Strategy on RDs of the NHS (see details in Acknowledgements).

In addition, five members of the RD Program of the Catalan Health Service, actively involved in the entire development and implementation process of the XUECs in Catalonia, also contributed in a second step by reviewing and validating the contributing aspects of the Catalan RDs Program, with no participation in the discussion about the identification of the strengths and weaknesses by the expert task force nor the recommendations drafted to improve or solve them.

### Questionnaire and focus groups

To capture the baseline opinions, explicit assessment and latent discourses of the participants, a questionnaire (Table 1) focused on identifying the strengths and weaknesses of the Catalonian healthcare system in RDs was filled out independently by all the task force members. The results obtained from the questionnaire sent to the experts were analyzed mostly qualitatively, since the questions included were open-ended. Quantitative analysis of those variables potentially assessed in grades or scales was also performed. Responses were collected and key topics identified were used as the input for the two sequential focus groups, in which interactive discussions were carried out. Overall, in these discussion forums all the participants contrasted their points of view and provided their approaches or perspectives by complementing the answers expressed in the initial survey, and also intended to delineate those dominant and underlying ideas and/or divergent viewpoints that felt outside the questionnaire. The first task force meeting was held to present the questionnaire’s initial information, and also to share experiences and identify positive aspects and areas for improvement. A first draft collecting all the main positive and negative points identified was elaborated as feedback document and sent to the experts. Subsequently, a second focus group was held to discuss about those items identified as the most common potential problems of the healthcare system regarding RDs care, for which the experts also tried to find potential solutions to improve or solve those identified weak areas that were reflected in a set of practical recommendations in the care of PLWRD.

**Table 1:** Questionnaire given to the panel of experts on RDs, prior to the focus groups

1	Considering the most recent advances regarding diagnosis and treatment of RDs, do you consider that the current RDs care model remains valid, or should it be updated in any of its areas?
2	As a care model, how would you asses the Network of Clinical Expert Units (Xarxa d’Unitats d’Expertesa Clínica or XUECs) in terms of:
	(a) Level of implementation?
	(b) Patient accessibility?
	(c) Number of accredited units?
	(d) Process of designation and continuous evaluation of the units?
	(e) Their level of development in research and education?
3	Considering the different phases of the care process for PLWRDs (diagnosis, treatment, follow-up and comprehensive care), in your opinion:
	(a) What are the key areas for improvement in each of these phases?
	(b) What are the most critical issues in the process?
	(c) What policies are being developed or implemented in Catalonia that you think should be highlighted and could be considered as *best practices*?
4	In terms of information and support for PLWRDs and their families, what actions would you highlight from their implementation, and what areas should be developed to improve the patient experience?
5	Beyond the clinical aspects, how would you rate the access of patients and families to other resources and/or social, healthcare and educational benefits, etc.?

### Analysis of the data

Because the initial questionnaire contained open-ended questions, the pre-existing or potential bias for selecting issues already expected was subsequently avoided or minimized in the focus groups. Focus groups were audiotaped and the transcriptions obtained from the two focus groups were processed by content analysis (to identify key topics) and discourse analysis (to examine the underlying narratives and communication patterns). In the second focus group meeting, the most important key words or topics, and potential solutions (numerically or proportionally more relevant in every context) from both questionnaires and the first focus group were revisited by the task force. Qualitative data was processed by transforming individual perspectives and potential strategies of action into collective key topics. In a consensus-building process, the experts refined the main suggestions and converted them into a final set of recommendations, which in turn were drafted based on the qualitative data gathered throughout the process. To conclude, the establishment of the strengths and weaknesses of the Catalan healthcare system for RDs and the final recommendations were reviewed and approved by all the members of the task force [25, 26].

## Results

Ten main areas of care for RDs in the Catalan Health Service in which strengths and weaknesses were selected by consensus by the acERca task force. The strengths and weaknesses are described in the tables, and the recommendations given by the experts intending to improve or solve the main problems identified are also provided.

### The healthcare model for care of RDs

The strengths and weaknesses identified about the healthcare model for RDs are illustrated in Table 2. The main recommendation of the participants was to invest more governmental resources to the XUECs accreditation and implementation processes. Creating and expanding more thematic areas of XUECs and having more personnel resources dedicated to increase and improve this process management and healthcare pathways was also requested. More resources dedicated to increase the administrative and technical staff in the Catalan Health Service could also contribute to improve the constant and regular evaluation of the accredited centers already belonging to the XUECs. It was considered relevant to promote campaigns aimed at professionals and patients for them to be aware of the existence of this RDs healthcare model and how it works.

**Table 2:** Strengths and weaknesses of the healthcare model for RDs

Strengths	Weaknesses
The Catalan Government healthcare model for the comprehensive care for RDs is currently valid and adapted to the needs of PLWRD and their families since its strategic framework is optimal and operational, and adequately reflects the ideal pathway to achieve a holistic care for RDs	The application and implementation of the aspects presented in the health model of care for RDs in Catalonia are still under development since they are not fully implemented in a daily basis
The healthcare system in Catalonia has specialized and accredited professionals since most patients diagnosed with RDs are already being assisted in national and international reference units of tertiary hospitals	In practice, not all patients still have convenient access to resources, professionals, and clinical protocols used in the reference centers for RDs (officially accredited as XUEC or not)
The Catalan Health Service counts with the counselling of the CAMM, a multidisciplinary and interdepartmental advisory commission that includes patient representation	The designation and implementation of every XUEC area is quite slow and the derivation system is not fully operative. Technical challenges causing this lacking operative system have been detected in the employment of a new information and communication technology (ICT) tool
XUECs are being accredited and implemented since 2015	Several thematic areas are still not covered by the present XUEC platforms
The identification of the reference centers that compose the different XUECs is carried out through a structured assessment procedure, which involves the Catalan Health Technology Assessment agency and a collegiate commission (of the Catalan Health Ministry and CatSalut) that includes patient representation	Difficulties for identifying experts in some areas are relatively common
A multidisciplinary team with demonstrated experience on RDs care is mandatory for the accreditation of a medical unit as a member of a XUEC	
At the time of the current study, nine XUECs had been already accredited, including RDs caused by cognitive and behavior impairment of genetic origin, hereditary metabolic diseases, renal disorders, neuromuscular disorders, epilepsy disorders, hemophilia and congenital coagulopathies, and immune-mediated disorders (inherited primary immunodeficiencies, autoinflammatory diseases and systemic autoimmune diseases, including pediatric rheumatic diseases)	
All the Catalan centers accredited for CSURs and ERN are members of the equivalent XUEC (when designated)	
XUECs designation has boosted the identification of PLWRD in the healthcare system and its codification with the Orphanet system	

### Coordination with primary HCPs and other tertiary and secondary hospitals

With regard to this subject, strengths and weaknesses are depicted in Table 3. The panel members recommended the creation of new standardized assistance processes to connect the already established XUECs with the primary healthcare system, other tertiary hospitals (not belonging to XUECs), and the secondary or proximity hospitals. The interconnection among physicians should improve and promote an official simplistic and easy referral process, starting by specific forms or templates for referring patients with a suspected or confirmed RD to the XUEC from the primary care system and also from other secondary and tertiary hospitals. In addition, the continuous collaboration and interaction in the comprehensive RDs care between the proximity centers and the XUEC centers is considered crucial for controlling PLWRD during the follow-up period.

**Table 3:** Strengths and weaknesses of the coordination of XUEC on RDs with other centers in the territory

Strengths	Weaknesses
Primary healthcare centers in Catalonia are well connected by an electronic network system (the HC3 platform) that provides details of visits and tests performed to the patient in all the centers of the public healthcare system	Although under development, no referral forms or templates to refer a patient with a suspected or confirmed RD from any primary or secondary care facility to the corresponding XUEC are available yet
	The network of XUEC HCPs (tertiary university hospitals) is not yet connected to the primary healthcare system or the remaining tertiary and secondary hospitals not belonging to the XUECs
	No specific budget is dedicated to reserve time into professionals’ agendas to coordinate and/or consult with other levels of care to decide a personalized treatment and/or how to schedule/combine next follow-up visits

### Access to and coordination with non-medical services of PLWRD

The strengths and weaknesses identified in this topic are illustrated in Table 4. The Government was found to be responsible for ensuring and facilitating patients’ access to medical/non-medical supporting services, usually required for continuous or prolonged periods, such as rehabilitation, psychological therapy, and social and educational resources. Therefore, to improve the connection between XUEC centers and these medical and non-medical resources/facilities in the territory, the expert panel suggests that a list of multidisciplinary professionals in charge in all XUEC hospitals, tertiary/secondary non-XUEC hospitals and other facilities, as well as agile access circuits, should be created in order to facilitate PLWRD and their families to reach the person of reference in every center involved in RDs healthcare.

**Table 4:** Strengths and weaknesses of the access to and coordination with non-medical services

Strengths	Weaknesses
The Catalan Health Service has a fairly good level of implementation of supportive rehabilitation and psychological services, and also social and educational support are available for the general population and people with physical or mental disabilities or special needs	Overall, PLWRD, even those controlled in XUEC reference centers, struggle to find official itineraries/pathways to access other (mainly local) health services such as continued rehabilitation or psychological support, and services from other governmental institutions, including those related to social and educational issues
	A lack of information about resources and access to them in the different governmental ministries (e.g. healthcare, social work, education) was identified

### The role of case manager in the network of RDs

The strengths and weaknesses of the existence of a case manager in the current XUEC system are listed in Table 5. The expert panel recognizes that increasing the time dedicated to case management and coordination of PLWRD, as well as hiring more trained personnel to develop case manager tasks, would solve (or at least improve) the detected problems regarding the lack of well-prepared case managers fully dedicated to RDs in the accredited XUEC centers. All the panel members agree that nurse practitioners or advanced practice registered nurses are the best professional profiles to cover the position of case manager for PLWRD with high level of complexity or multidisciplinary requirements.

**Table 5:** Strengths and weaknesses of the case manager role

Strengths	Weaknesses
The functions of case managers for RDs are well established by the Catalan Health Service as the person in charge of the coordination and organization of the care processes (between different hospital departments and between hospital and non-hospital medical and non-medical facilities), and as the person providing information and support to patients and their families regarding these processes	The figure of case managers is not well-known by professionals not familiarized with XUECs, even by those working in hospitals
For every designated unit belonging to a XUEC, the Catalan Health Service considers mandatory, as a commitment, the existence of a case manager	Not all hospitals or units with accredited XUECs have case managers fully dedicated to RDs since in many centers there is no governmental or hospital budget dedicated to this purpose
Most of the case managers are nurses specialized and trained in RDs	There is a lack of nurses in the Spanish and Catalan health systems. This fact does not favor the exclusive dedication of nurses to the specific field of RDs

### Genetic diagnosis of PLWRD

The strengths and weaknesses of the genetic diagnosis of RDs in Catalonia are depicted in Table 6. Based on the extension of Genetics services and units, the experts agree that there is a fairly good genetic healthcare, although insufficient due to the absence of proper postgraduate and specialized training in clinical genetics and genetic counseling by physicians and clinical scientists. In addition, despite the high availability of genetic testing, the gold standard of DNA sequencing based on singleton whole exome sequencing (WES) and trio-WES is not fully implemented.

**Table 6:** Strengths and weaknesses of the genetic diagnosis of PLWRD

Strengths	Weaknesses
The Catalan and Spanish health systems provide universal and public healthcare to all the inhabitants. Therefore, all the required diagnostic tests are provided equally to everyone. In this regard, the newborn screening program (NSP) currently covers seven diseases in the national mandatory portfolio for all the 17 autonomous regions, but the number of diseases/genes differs among regions. In Catalonia, the current number of RDs included in the NSP program is 25, with 15 additional neonatal conditions detectable in a secondary panel by an abnormal biomarker	There is lack of uniformity among screening programs regarding the number of RDs included, both between EU countries and between regions of Spain. This means there is inequity between citizens depending on where they live
The Catalan Health Service provides genetic medical care through hospital Genetics services and units of university hospitals distributed throughout the territory, with a high capacity to offer genetic and genomic tests	The number of reference centers performing genetic testing based on exome/genome sequencing and optical mapping, and computational and analytical capabilities for patients with suspected RDs is insufficient. The availability of genomic sequencing is still expensive and scarce. Modern genomic technologies, and more specifically DNA sequencing current gold standards WES and WES-trio, have not been fully established for RDs genetic testing
Attending staff in genetics services has training and experience in the field of human and clinical genetics, but not obtained in a regulated manner. Medical staff attending medical consultations has clinical training in pediatrics, internal medicine, or obstetrics and gynecology. The laboratory staff has either laboratory medicine specialization or graduate training in the field of biological sciences and a master’s degree in genetics or biomedicine	Because clinical genetics is not an officially recognized healthcare specialty in Spain, there is no formal training in clinical genetics for both physicians and laboratory scientists. Training in genetic counselling is based on a few university master’s degrees. This absence of official recognition of clinical genetics specialty precludes the offer of positions in public reference university hospitals for physicians, clinical scientists, and genetic counselors

The forum experts agree in that a constant communication is required (or should be improved) between XUEC (mostly pediatric) centers and Child Development and Early Care Centers (Centres de Desenvolupament Infantil i Atenció Precoç or CDIAP) and Child and Adolescent Mental Health Centers (Centre de Salut Mental Infantil i Juvenil or CSMIJ) after a genetic diagnosis is achieved.

### Patients with undiagnosed RDs

Strengths and weaknesses of the topic about undiagnosed patients with suspected RDs are listed in Table 7. The expert panel recognized the need for medical education of primary HCPs as well as pediatricians and physicians in secondary (local) and tertiary hospitals in order to facilitate the agile identification of the specialty branch and the appropriate XUEC (if implemented) to which the patient must be referred. Once the patient has reached the reference center, all the required diagnostic tests must be performed. When the patients remain “undiagnosed”, it is important to properly label them using terms such as “undiagnosed”, “unclassified” or “undifferentiated” RDs, so that it could be identified into the healthcare system and by any HCP, either in primary or specialized care.

**Table 7:** Strengths and weaknesses regarding undiagnosed patients with a suspected RD

Strengths	Weaknesses
The Catalan Health Service RDs strategy aims to guarantee that undiagnosed patients have access to specific clinical and genetic units to receive an appropriate diagnosis procedure	About half of patients remain without a definite diagnosis, which prevents access to targeted therapies
	Most primary care physicians and pediatricians, but also professionals working at secondary or tertiary hospitals, do not know to which specialists undiagnosed RDs patients must be referred for further clinical and genetic assessment
	There is a lack of recognition of patients with potential undiagnosed RDs (even in secondary and tertiary hospitals), and subsequently, an insufficient referral process to reference centers on RDs, which is crucial for these patients to obtain the best diagnostic and therapeutic approaches currently available

### Treatments for PLWRD

Strengths and weaknesses of this issue are illustrated in Table 8. The participants consider that the emergence of new advanced and innovative drugs is transforming the treatment paradigm for many RDs. Furthermore, they expressed their concern about the delay in access to innovation in recent years in Spain, which is particularly worrisome when it comes to therapies that provide therapeutic value in areas or diseases with few or no treatment options. The governmental resolution time, depending on the Spanish Ministry of Health, for the approval and funding of new orphan drugs is generally considered too long. The task force also agrees that the Spanish and Catalan governments should dedicate more funding to effectively expand the operational capacity of XUEC centers regarding treatment prescription in patients with diagnosed and undiagnosed RDs. This should also include the access to off-label and experimental medications to patients in whom those drugs can be used according to the current scientific knowledge, especially in those RDs without any approved therapeutic alternative. Because off-label indications are not formally funded by the public healthcare system in Spain, the indication and prescription of these drugs for non-authorized indications is charged to the reference prescribing center. Consequently, since this system policy economically impacts the own reference center, an update in the economical distribution of the treatment model is urgently required. There is also consensus on that indicating and prescribing certain orphan and complex (usually very expensive) drugs should be done in reference centers officially designated as XUECs. This issue is currently under consideration in Catalonia. The participants also requested that the current system should measure clinical outcome indicators of patient response to orphan drugs to not perpetuate useless but expensive therapies. The Catalan Heath Service is already systematically collecting the outcome variables of some authorized hospital medications. However, this should be further explored and implemented.

**Table 8:** Strengths and weaknesses of treatment availability for PLWRD

Strengths	Weaknesses
The Catalan Health Service allows the prescription and administration of approved or authorized orphan drugs for certain RDs since the payment is centralized in a governmental budget. All the reference centers belonging to the XUECs are aware and are able to prescribe authorized therapies for those recognized RDs	Not all orphan and complex drugs prescriptions are officially assigned by the Catalan Government to specific reference centers, since they can be still prescribed in a broader range of hospitals
Off-label indications are allowed and can be prescribed and administered, usually in the reference centers	Off-label indications for non-authorized drugs are difficult to obtain from the reference center itself (due to management or pharmacy department policies of some centers) because there is not a centralized governmental funding for this type of drugs and the budget in these situations is charged to the prescription center (and not to the referral hospital or to common governmental funds)

### Referring process, continuous follow-up and transition from pediatric to adult centers of PLWRD

Strengths and weaknesses of this topic are illustrated in Table 9. The participants suggest the need to comply the described Catalan RDs model with governmental measures to enforce the current rule of referring all patients with a confirmed or suspected RD to the accredited reference centers of the XUECs. This would legitimate the healthcare plan for RDs and would facilitate the best diagnosis, treatment and control in all PLWRD. The experts also recommend forcing better coordination and collaboration of XUEC specialists with secondary or tertiary proximity hospitals, and even with primary care facilities, in order to minimize the number of follow-up visits. This would ensure a minimal disruption of the daily routine of PLWRD, such as missing school attendance or workdays for pediatric and adult patients, respectively. The implementation of telemedicine and other new communication technologies can also be useful to accomplish this goal, as well as any means of support for patients that need to travel to a XUEC hospital due to follow-up visits. As these diseases are usually diagnosed in childhood, it is also important to help and accompany patients during the transition from childhood/adolescence to adulthood. Common transition protocols and guidelines are required for all centers belonging to the same XUEC.

**Table 9:** Strengths and weaknesses of the referring, follow-up, and transition (from pediatric to adult centers) processes for PLWRD

Strengths	Weaknesses
Referring patients with a confirmed or a suspected RD from healthcare centers in the territory to a XUEC center is a mandatory action according to the Catalan Health Service policy for RDs	Not all healthcare professionals in tertiary or secondary (proximity) hospitals refer patients with confirmed or suspected RD to the XUEC centers (this is known because there is consensus on that many patients still ask for opinion to XUEC centers without their local physician support)
Within the Catalan Health Service, the XUEC model allows the diagnosis, treatment indication and follow-up in those accredited reference centers. However, for follow-up, it is highly recommended a shared and continuous control between XUEC centers and other tertiary or secondary (local) hospitals (ideally involving also primary care facilities)	PLWRD and their families/caregivers living far away from the XUEC reference hospital usually spend too much travel time and frequently loose school days and/or workdays, with no economic compensation
The transition process from pediatric to adult centers when children with RDs reach adulthood is a requirement for all XUEC centers. Units or centers with pediatric and adult XUEC membership are already correctly using transition processes	Homogeneous guidelines and protocols for transition are still needed for those non-XUEC centers without continuity in attention of patients with different ages (mainly in those with only pediatric or adult RDs units)

### Research and education for professionals involved in the care of PLWRD

Strengths and weaknesses regarding professionals involved in the PLWRD care are depicted in Table 10. The experts recommend that more and better educational resources should be available to general practitioners and primary care pediatricians, but also to professionals working at secondary and tertiary (non-accredited as XUEC) centers, in order to enable them to efficiently suspect a RD and refer the patient to the appropriate reference center. Educational meetings between XUEC and non-XUEC professionals would improve knowledge on the Catalan healthcare model for RDs and would encourage sharing patient management by involving both proximity and highly specialized healthcare resources.

**Table 10:** Strength and weaknesses of the education provided to professionals involved in PLWRD care

Strengths	Weaknesses
All the centers officially designated as XUEC do have accredited research and educational merits, since these were requirements to be officially designated as a XUEC member	Investment to enhance research and to increase the number of expert professionals on RDs, involved in attending, research and educational tasks, is insufficient
	Apart from outpatient clinical visits during working hours, most of the remaining tasks rely on the free time of the healthcare professionals and investigators
	Specific educational meetings about RDs and the available circuits within the healthcare system for PLWRD directed to primary care physicians, pediatricians, specialists from secondary and tertiary (proximity) hospitals, and nurses and other professionals involved in the attention of PLWRD are still needed to emerge or improve

### Relationship with associations of PLWRD and their families (patients’ advocacy)

Strengths and weaknesses regarding the current situation of patient associations and families of PLWRD are depicted in Table 11. The experts consider crucial for the well-being of patients, their families, and caregivers to provide them educational diagnosis tools to help identifying RDs as well as support them during the disease course and treatment management in order to alleviate the physical, mental/psychological and economic burden associated to most RDs. There is a need of assistance home tools and ensuring access to rehabilitation, prosthetics, and other special resources (depending on the disease). In addition, better coordination between the centers belonging to the XUECs and mental, social, and educational resources/facilities (such as CDIAP and CSMIJ) is required. To summarize all the needs identified, it would be desirable to have common guidelines addressed to patients and their families specific to their RD (or RDs group) from the corresponding XUECs. These protocols should include therapeutic management of RDs and information about medical and non-medical resources involved in their care and their structural connections and functions. It is considered important to incorporate or increase the participation of patient associations to the different decision-making forums. The expert task force also recommended promoting the collaboration between public and private initiatives to enhance good quality research about diagnosis, management, and treatment strategies for RDs.

**Table 11:** Strengths and weaknesses of the relationship with patient associations and families of PLWRD

Strengths	Weaknesses
Patient associations for new RDs have been increasingly constituted in Catalonia and Spain	Currently, access and economical support to services considered essential for PLWRD, such as continued physiotherapy, rehabilitation, and psychological, or speech therapy, among others, are totally or in part covered by families, or patient associations (or their federations)
Federations of patient associations are present at Spanish and Catalan levels as FEDER and FECAMM, respectively. These federations are playing an active role at a governmental level (in the Spanish and Catalan health systems)	There is a long road ahead for patient associations to be part of the different existent decision-making forums
Patient advocacy groups are beginning to be part of decision-making forums since there is an agreement that patients can play a key role in contributing to improve or influence changes in health and social policies	There is still insufficient professionalization of patient associations and their representatives and a perception of lack of confidence in patient representation from the administration and other groups of professionals involved in RDs healthcare plans

## Discussion

Catalonia has a solid healthcare system with a strong interest in RDs since the creation of the CAMM in 2009 and the development in 2010 of the Catalan model on RD care based on the XUECs [7, 18], the only specific model with accredited reference centers dedicated to RDs in Spain to date. The XUECs structure was based in grouping RDs by thematic areas, following a similar division followed by the ERN strategy [27]. However, differently to ERNs, in which the whole hospital is usually labeled as reference center for all diseases included in the ERN, in the case of XUECs, all the subgroups of diseases included in a single XUEC were also evaluated separately for every applicant center, and the hospital is only accredited in those clinical units for which the application criteria in every disease group are fulfilled. Although the majority of RDs in the XUECs have been and are being distributed according to the ERNs division, several specific conditions or groups of diseases of the XUECs have been grouped differently than in ERNs. For instance, diseases included in ERN of Rare Immunodeficiency, Autoinflammatory and Autoimmune Diseases (RITA) and ERN on Connective Tissue and Musculoskeletal Diseases (ReCONNET) have been assembled in the XUEC of Immune-mediated Diseases, which in turn has been divided in three main areas: Primary Immunodeficiencies, Autoinflammatory Diseases, and Systemic Autoimmune Diseases (including the Autoimmune Diseases and Vasculitis subareas) [28]. In Spain, to achieve any ERN membership, the applicant center must have been previously accredited with a CSUR in similar RD areas. In Catalonia, the application and audit actions for XUEC, CSUR and ERN accreditations are separate processes. Of note, after the second ERN call in 2021 and by 2024, Catalonia was the Autonomous Region in Spain with the highest number of designed Spanish and European reference centers, and 117 (31.9%) of the total 367 CSUR members and 55 (50.5%) of the total 109 ERN Spanish members corresponded to Catalan centers [21, 29, 30]. In Catalonia, 98 (88.3%) of the 111 Catalan CSURs on RDs, and 33 (60%) of de 55 XUECs’ Units were also ERN full members, respectively [20, 28, 30]. The higher overlap between CSUR and ERN compared to XUEC is explained by the fact that a CSUR designation is mandatory to participate in ERN calls. Nevertheless, almost all the Catalan centers accredited as ERN full members are also members of the corresponding XUEC.

Despite the significant effort done by the Catalan Health Service in the creation and implementation of the XUEC model for RDs care in Catalonia, which has been identified by the panel of experts in this study as part of the strengths of the Catalan Government strategy for RDs, several weaknesses were detected. The problems found in the current XUEC model for RDs were grouped in ten main areas: (1) the healthcare model for RDs; (2) coordination with primary healthcare centers and other tertiary and secondary hospitals; (3) access to and coordination with non-medical services; (4) the role of case manager in the XUEC; (5) genetic diagnosis; (6) undiagnosed patients; (7) treatments; (8) referring process, continuous follow-up, and transition from pediatric to adult centers; (9) research and education for professionals; and (10) associations of PLWRD and their families.

Although all the identified aspects as weaknesses of the XUEC strategy in Catalonia had been contemplated as key points by the Catalan Health Service, two common feebleness were found in most of them: deficiencies in the operability (or real implementation) and the limited allocation of crucial resources (mostly in administrative, technical, and medical personnel, therapies implementation, and medical and non-medical services) for the proper function of the entire healthcare system for PLWRD. Table 12 summarizes the 10 key recommendations to improve or solve the care of PLWRD in Catalonia.

**Table 12:** The ten key recommendations to solve or improve the care of PLWRD

Identified weaknesses	Recommendations
1. The health model for care of RDs	More governmental resources to the XUECs accreditation and implementation processes, and also for creating and expanding more thematic areas of XUECs, are needed. More personnel resources dedicated to increase and improve the administrative process should be provided. New ICT tools should be applied to start the derivation process of patients with a confirmed or suspected RD to the XUECs’ centers
2. Coordination with primary HCPs and other tertiary and secondary hospitals	New standardized assistance pathways should be created to connect the already established XUECs with all the HCPs involved in RDs care (primary health care system, secondary and tertiary hospitals, mental health services, etc.)
3. Access to and coordination with non-medical services of PLWRD	The connection between XUEC centers and medical and non-medical services (e.g. rehabilitation, psychological therapy, and social and educational resources) in the territory should be improved to ensure and facilitate patients’ access to these resources. A multidisciplinary contact network of professionals working in these facilities should be created
4. The role of the case manager in RDs care	More resources should be dedicated to the existence of well-prepared case managers fully dedicated to RDs in the accredited XUEC centers
5. Genetic diagnosis	Permanent communication should be established between XUEC (mostly pediatric) centers and external (mostly neurologic and psychiatric) facilities after a genetic diagnosis of a RD is achieved
6. Undiagnosed patients with a suspected RD	Medical education and (frequently updated) information on the RDs care model should be provided to primary HCPs as well as pediatricians and physicians of secondary and tertiary (local) hospitals with the aim of facilitating early access of patients with suspected RDs to appropriate XUEC centers to perform all the required tests
7. Treatments implementation of orphan drugs	The governmental resolution time for the approval and funding of new orphan drugs should be reduced. More funding should be invested to expand the operational capacity of XUECs with regard to treatment prescription in patients with diagnosed and undiagnosed RDs. This should also include the access to off-label and experimental medications for non-diagnosed patients or patients with established RDs
8. Referring, follow-up and transition (from pediatric to adult centers) processes for PLWRD	Official measures from the Catalan Government to enforce the current rule of referring all patients with a confirmed or suspected RD to the accredited reference centers of the XUECs would legitimate the health plan for RDs and would foster the best options for diagnosis, treatment and control of all PLWRD. A better coordination and collaboration of XUECs specialists with secondary or tertiary proximity hospitals, and with primary care facilities, as well as the next implementation of telemedicine visits, would minimize the number of visits during patients’ follow-up
9. Research and education for professionals involved in the care of PLWRD	More and better educational resources and information about the Catalan healthcare model for PLWRD and the functional structure of the XUECs should be available to primary care physicians, pediatricians, and professionals working at secondary and tertiary (proximity) centers
10. Associations of PLWRD	Having available educational diagnosis tools to identify RDs, and receiving medical and psychological support during the disease course and treatment in order to alleviate physical, emotional and economic burden of an RD diagnosis and follow-up are considered crucial factors to improve for the wellbeing of PLWRD, their families and caregivers. To promote common therapeutic guidelines, to provide official information about structural connections and functions of the different resources/facilities in every XUEC (addressed to PLWRD and their families), and to incorporate patient associations to the decision-making forums are also important aspects to be consolidated

The improvement of care for PLWRD and their families has led, in recent years, to the development of institutional plans regarding RDs care and implementation of multidisciplinary and collaborative models for providing better attention and management of patients and families. In Spain, the strategies of political actions at different territorial levels have led to stablish European (ERNs in the EU) [10, 21–24], national (CSURs in the Spanish NHS) [6, 19–22], and regional actions (XUECs in Catalonia) RDs networks [7, 18]. In this regard, the Catalan (XUEC) model is an example of the impact of the ERN structure at regional level that goes beyond the initial ERN objectives since this local healthcare system also addresses practical and already operative daily care aspects by facilitating the referral, visit and follow-up of patients in the designed reference centers, which definitely makes patient journey shorter, even in comparison with CSURs (that serve mostly as a consultation system, since patients have to be treated in their local centers) and ERNs (that do not influence the direct assistance or treatment of patients in expert centers of other countries to date). The XUEC system is already integrated and functioning as part of the Catalan autonomous healthcare system in Spain, and also represents a clear example of a regional action in connection with both the Spanish Government and the healthcare actions originated in the EU. Because most designed centers are also members of the ERNs, these expert centers are also facilitating ERN goals at regional level, such as the establishment of quality assurance models, referral pathways for PLWRD, and programs for undiagnosed diseases, or other strategies for improving data management and dissemination of information among patients and healthcare professionals. Since June 2023, all these (and other) measures are currently being evaluated by the EU for developing models and recommendations to integrate ERNs tasks into the healthcare systems of the member states by the Joint Action on Integration of ERNs into the National Healthcare Systems (JARDIN) [31, 32].

## Conclusions

Based on the criteria of equity and equality for which a public healthcare must be guaranteed to all citizens, PLWRD have also the right to access to all the available resources in a way that is as agile and equal as for other patients. The expert participants considered that the current Catalan healthcare model for RDs consists of a pioneer and advanced plan, unique in Spain, consisting of a functional network of qualified reference centers (XUECs), following the European model of the ERN thematic areas. Compared to the ERN system, this XUEC model has the advantage of being operational since it includes patient visits for diagnosis, treatment and follow-up in these reference centers. At the same time, XUEC centers are connected with other hospitals and non-medical facilities in the Catalan territory. However, the main weaknesses identified in the XUEC model included different items, many of which are related to an insufficient investment in the administrative personnel in charge of the XUECs accreditation and implementation processes, including the coordination of those XUEC centers with other centers and health services in the territory. The derivation system to XUECs’ centers was considered non-fully operative due to the lack of resources and because of the technical challenges of implementing a new ICT tool, which is expected to become available and integrated into the operative systems of the majority Catalan healthcare centers in the near future. Additional problems were also detected at different stages of the patient journey and the comprehensive RDs care, such as difficulties in accessing to specific reference centers and non-medical services, finding non-medical professionals with knowledge in diagnosing and managing PLWRDs, as well as limited access to some orphan treatments and follow-up visits in the specialized centers. The transition of patients from pediatric to adult centers, research and educational programs for healthcare professionals, and support to RD patient associations were other challenges detected. Although the insufficient administrative support and governmental investments were identified as major factors for the Catalan healthcare for RDs operation, it is also important to resolve or improve other aspects such as the prioritization of RDs in the hospitals’ and other facilities’ own budget management, the need of new ICT tools (currently limited) since they may potentially facilitate many communication processes, and the requirement to encourage a culture of cooperation among professionals. In comparison to other governments, in which dedicated personnel and support for implementing similar systems are often lacking, the efforts made by the Government of Catalonia are commendable and demonstrate a strong commitment to progress in the RDs field. However, in this regard there is always room for improvement and, therefore, it is essential to promote and support new governmental political and administrative decisions. It is also remarkable that most of the strategic points of the Catalan Health Service for RDs are aligned with those goals proposed by the EU in which ERNs are expected to be connected in the future with all healthcare systems of the member states. Finally, the proposed recommendations resulting from this participative study aim to help strengthen the current Catalan healthcare strategy on RDs to facilitate and improve the quality of life and survival of PLWRD in Catalonia, as well as they can assist RDs in other regions and nations worldwide.

## Data Availability

Data sharing is not applicable to this article as no datasets were generated or analyzed during the current study.

## Abbreviations

CSUR: Centros, Servicios y Unidades de Referencia (Reference Centers, Services and Units)
CAMM: Comissió Assessora de Malalties Minoritàries (Advisory Commission on Rare Diseases)
CDIAP: Centres de Desenvolupament Infantil i Atenció Precoç (Child Development and Early Care Centers)
CSMIJ: Centre de Salut Mental Infantil i Juvenil (Child and Adolescent Mental Health Centers)
EMA: European Medicines Agency
ERN: European Reference Network
EU: European Union
FECAMM: Federació Catalana de Malalties Minoritàries (Catalan Federation of Rare Diseases)
FEDER: Federación Española de Enfermedades Raras (Spanish Federation of Rare Diseases)
ICT: Information and Communication Technology
JARDIN: Joint Action on Integration of ERNs into the National Healthcare Systems
HCP: Healthcare provider
NHS: National Health System
NSP: Newborn Screening Program
PLWRD: Persons living with a rare disease
RD: Rare disease
RITA: Rare Immunodeficiency, Autoinflammatory and Autoimmune Diseases
ReCONNET: Connective Tissue and Musculoskeletal Diseases
WES: Whole exome sequencing
XUEC: Xarxa d’Unitats d'Expertesa Clínica (Network of Clinical Expert Units)
